# Traditional Chinese medicine promotes bone regeneration in bone tissue engineering

**DOI:** 10.1186/s13020-022-00640-5

**Published:** 2022-07-20

**Authors:** Zheng-Rong Gao, Yun-Zhi Feng, Ya-Qiong Zhao, Jie Zhao, Ying-Hui Zhou, Qin Ye, Yun Chen, Li Tan, Shao-Hui Zhang, Yao Feng, Jing Hu, Ze-Yue Ou-Yang, Marie Aimee Dusenge, Yue Guo

**Affiliations:** 1 Department of Stomatology, The Second Xiangya Hospital, Central South University, 139 Renmin Middle Road, Changsha, 410011 Hunan China; 2 Department of Endocrinology and Metabolism, Hunan Provincial Key Laboratory of Metabolic Bone Diseases, National Clinical Research Center for Metabolic Disease, The Second Xiangya Hospital, Central South University, Changsha, Hunan China

**Keywords:** Traditional Chinese medicine, Bone tissue engineering, Bone regeneration, Scaffolds, Osteogenesis

## Abstract

Bone tissue engineering (BTE) is a promising method for the repair of difficult-to-heal bone tissue damage by providing three-dimensional structures for cell attachment, proliferation, and differentiation. Traditional Chinese medicine (TCM) has been introduced as an effective global medical program by the World Health Organization, comprising intricate components, and promoting bone regeneration by regulating multiple mechanisms and targets. This study outlines the potential therapeutic capabilities of TCM combined with BTE in bone regeneration. The effective active components promoting bone regeneration can be generally divided into flavonoids, alkaloids, glycosides, terpenoids, and polyphenols, among others. The chemical structures of the monomers, their sources, efficacy, and mechanisms are described. We summarize the use of compounds and medicinal parts of TCM to stimulate bone regeneration. Finally, the limitations and prospects of applying TCM in BTE are introduced, providing a direction for further development of novel and potential TCM.

**Graphical Abstract:**

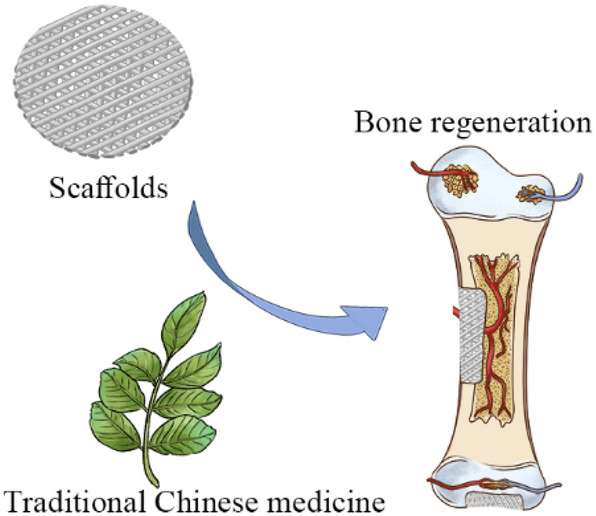

## Traditional Chinese medicine (TCM)

### Role of TCM

TCM comprises natural products and extracts derived from herbs, animals, and minerals with effective bio-functions for maintaining health and treating disease. During decades, its use has been widespread globally [1–3]. As early as the Eastern Han Dynasty, the classical text on Chinese Medicine, *Shen Nong’s Materia Medica* (Shen Nong Ben Cao Jing), recorded the use of TCM in the treatment of diseases. Nowadays, TCM has been shown to play a crucial role in the prevention and management of diseases, such as cardiovascular disease, cancer, and diabetes [4–6]. TCM was considered a “miracle” drug for certain major diseases, such as artemisinin in malaria and arsenic trioxide in acute promyelocytic leukemia [7, 8]. Therefore, further understanding and expanding the use of TCM is necessary for continued developments in the field.

### Classification of TCM

The classification of TCM is sophisticated and complex. From the ancient books and Pharmacopoeias, such as *Shen Nong’s Materia Medica* [9], *Compendium of Materia Medica* (Ben Cao Gang Mu) [10], *Yellow Emperor’s Inner Canon* and *Treatise on Cold Damage*, to the latest Chinese Pharmacopoeia [11, 12], the classification standard of TCM are still difference. And some of the scholar different from the traditional classification in China, some scholars divided TCM into Alkaloids, Terpenoids, Flavonoids, Volatile Oils etc. based on the active components (Table 1). The classification of TCM in this review is based on the active components reported for application in bone tissue engineering (BTE) that promote bone formation, including flavonoids, alkaloids, glycosides, terpenoids, and polyphenols, among others. And the drug component has been shown in Table 2. The classification of TCM is significant for guiding clinical application and avoiding instability and unsafety caused by improper combination [13].

**Table 1:** The classification standard of TCM

Classification standard	Category	References
*Shen Nong’s Materia Medica*	Top-grade	[9]
	Medium-grade	
	Low-grade	
*Compendium of Materia Medica* (Ben Cao Gang Mu)	Sources, habitats, colors, parts of plants, and how they were collected, processed, and selected for prescriptions	[10]
	Four fundamental characters: cold, cool, warm, and hot	[27]
	Five fundamental tastes: salty, sour, bitter, sweet, and pungent	
	Four toxic states: toxic, nontoxic, very toxic, and slightly toxic	
	12 meridians: bladder, spleen, large intestine, stomach, small intestine, liver, cardiovascular, heart, kidney, gallbladder, pericardium and san jiao	
*Yellow Emperor’s Inner Canon*	Herb-derived medicine, animal-derived medicine and mineral-derived medicine	[11, 12]
*Treatise on Cold Damage*		
Chinese Pharmacopoeia		
Active components	Alkaloids	[28–30]
	Terpenoids	
	Flavonoids	
	Volatile Oils	
	Lignanoids	
	Coumarins	
	Quinones	
	Phenols	
	Glycosides	
	Saponins	
	Stilbenes	
	Phenols	
	Esters	

**Table 2:** The basic information of TCM

Category	Components	Structural forms	Molecular weight	Source	Main function	References
Flavonoids	Icariin		676.67	*Epimedium* species	Treatment of fractures, joint disease, and gonadal dysfunctions	[31, 32]
	Icaritin		386.4	*Epimedium* species	Osteoprotective effect, neuroprotective effect, cardiovascular protective effect, anti-cancer effect, anti-inflammation effect, and immune-protective effect	[33]
	Hydroxy safflower yellow A		612.53	*Safflower*	Cardiovascular protection, coronary heart disease treatment and capillary angiogenesis, blood circulation and dispersing blood stasis	[34–38]
	Xanthohumol		354.4	*Humulus lupulus*	Stimulate osteogenic differentiation, anti-inflammatory, and inhibits osteoclastogenesis	[39–41]
	Kaempferol		286.23	*Kaempferia galangal*	Osteoporosis, diabetes, obesity, immune regulation, antiviral, and antidepressant treatments	[42–45]
	*Cuscuta chinensis* Lam.	Not applicable (NA)	N/A	*Kaempferia galangal*	Osteoporosis treatment	[45]
	Baicalin		446.36	*Radix Scutellariae*	Antioxidant, antiapoptotic, and immunoregulatory activities with minimal side-effects	[46, 47]
	Baicalein		270.24	*Radix Scutellariae*	Antioxidant, antiapoptotic, and immunoregulatory activities with minimal side-effects	[46, 47]
	Naringin		580.53	Tomatoes, grapefruits, and many other citrus fruits	Anti-inflammatory, antiapoptotic activities, and have therapeutic potential cancer, cardiovascular disease, diabetes, and oral disease	[48–50]
	Hesperetin		302.28	Chenpi	Antioxidant, anti-inflammatory, and anti‐carcinogenic effects	[51]
	Quercetin		302.24	*Quercetum*, fruit and vegetables	Anti-inflammatory, anti-viral, anti-oxidant, anti-cancer properties, osteogenesis and angiogenesis	[52, 53]
	Silymarin		482.44	*Silybum marianum*	Hepatoprotective effects, anti-viral, anti-Parkinson, anti‐Alzheimer effects, anti-cancer and anti‐inflammatory	[54, 55]
Alkaloids	Tetrandrine		622.76	*Stephania tetrandria*	Anti-inflammatory, immunosuppressant, anti-allergic effects, anti-oxidant, anti-diabetic and anti-microbial	[56, 57]
	Berberine		336.36	*Rhizoma coptidis*	Diabetes, anti-inflammation, anti-cancer therapies, lowing of blood lipids and promote bone formation	[58–60]
Glycosides	Ginsenoside Rg1		801.01	Ginseng	Cell proliferation and differentiation, anti-apoptosis, and anti-inflammation	[61]
	Ginsenoside Rb1		1109.31	Ginseng	Osteogenesis	[62]
Terpenoids	Ursolic acid		456.70	Fruits and vegetables	Anticancer, antioxidant, and other pharmacological effects	[63]
Polyphenols	Resveratrol		228.25	*Veratrum grandiflorum* O. Loes	Mediating inflammation, tumerogenesis, and cardioprotective effects	[64–66]
	Curcumin		368.39	*Curcuma long* L.	Anti-oxidant, anti-inflammatory, and anti-cancer effects	[67]
	Epigallocatechin gallate		458.38	*Camellia sinensis* L.	Anti-oxidant and anti-inflammatory effects	[68]

## BTE

Bone remodeling and regeneration are continuous and dynamic physiological processes regulated by two cellular mechanisms, namely bone formation and resorption [14]. The loss of bone tissue can occur following an accident, trauma, cancer, and congenital malformation. Although bone remodeling and regeneration is a lifelong process, a bone defect, especially A large one, severely affects the function of the defunct area and quality of life due to the limited self-repair capacity of bone tissue and some inevitable side effects after surgery [15, 16]. Currently, the clinical “gold standard” for bone transplantation and reconstruction is autogenous bone grafts; however, the risk of donor site morbidity, limited graft supply, and bone formation delay must be seriously considered [17, 18]. To address naturally arising difficulties, BTE has been applied to provide a cell friendly microenvironment for defect repair and tissue regeneration. BTE is an interdisciplinary field that combines the principles of engineering and biology to develop biological substitutes for restoring, maintaining, or improving bone tissue function [19, 20]. BTE has been applied in the treatment of large sections of bone tissue are absent, including traumas, bone cancer tumor resection, congenital malformation, and debridement of infected bone tissue [21]. The ideal characteristics of BTE including non-immunogenic, biocompatibility, controllable, readily available and have the mechanical properties similar to the natural tissue material, and possess suitable structure, architecture, and pore sizes for cells survival and activity [22, 23]. The advent and development of BTE brought about promising approaches for bone regeneration by ways of including the three key factors, namely (1) cells, (2) scaffolds, and (3) growth factors [24–26].

### The application of TCM in BTE

The integrate TCM with BTE has a unique advantage in bone regeneration. Oral administration is the typical route for drug delivery, but the drawbacks such as first-pass metabolism would reduce the drug efficacy [69], while the topical delivery would prevention of first pass effect by liver and gut enzymes [70, 71]. To achieve successful and satisfactory therapeutic results, oral delivery requires overcoming the challenges by increasing the permeability of the intestinal epithelial membrane, inhibiting the degrading enzymes or protecting therapeutic by encapsulation [72], while the topical delivery can avoid this problem. Besides, the side effects of large doses of drugs have not been fully elucidated. At present, some drugs, such as BMP-2 and PTH, can be combined with BTE to promote bone defect healing. However, this may lead to extensive spread and subsequent accumulation in different organs, and further induce negative systemic side effects. Meanwhile, the production of these medicines are expensive [73]. Although the systemic administration of TCM shows low toxicity and side effects, it usually takes effect slowly. Therefore, regional TCM may provide a suitable alternative to TCM therapy. By integrating TCM with BTE, the BTE could act as a delivery carriers, and the topically-applied drug cannot only reach the interior target tissue with a greater bioavailability, but also prolong residence time as well as sustain drug release [74]. At the same time, TCM combined with BTE can improve its mechanical properties, such as Young’s modulus, compressive strength, hydrophilicity [75, 76].

**Fig. 1:**
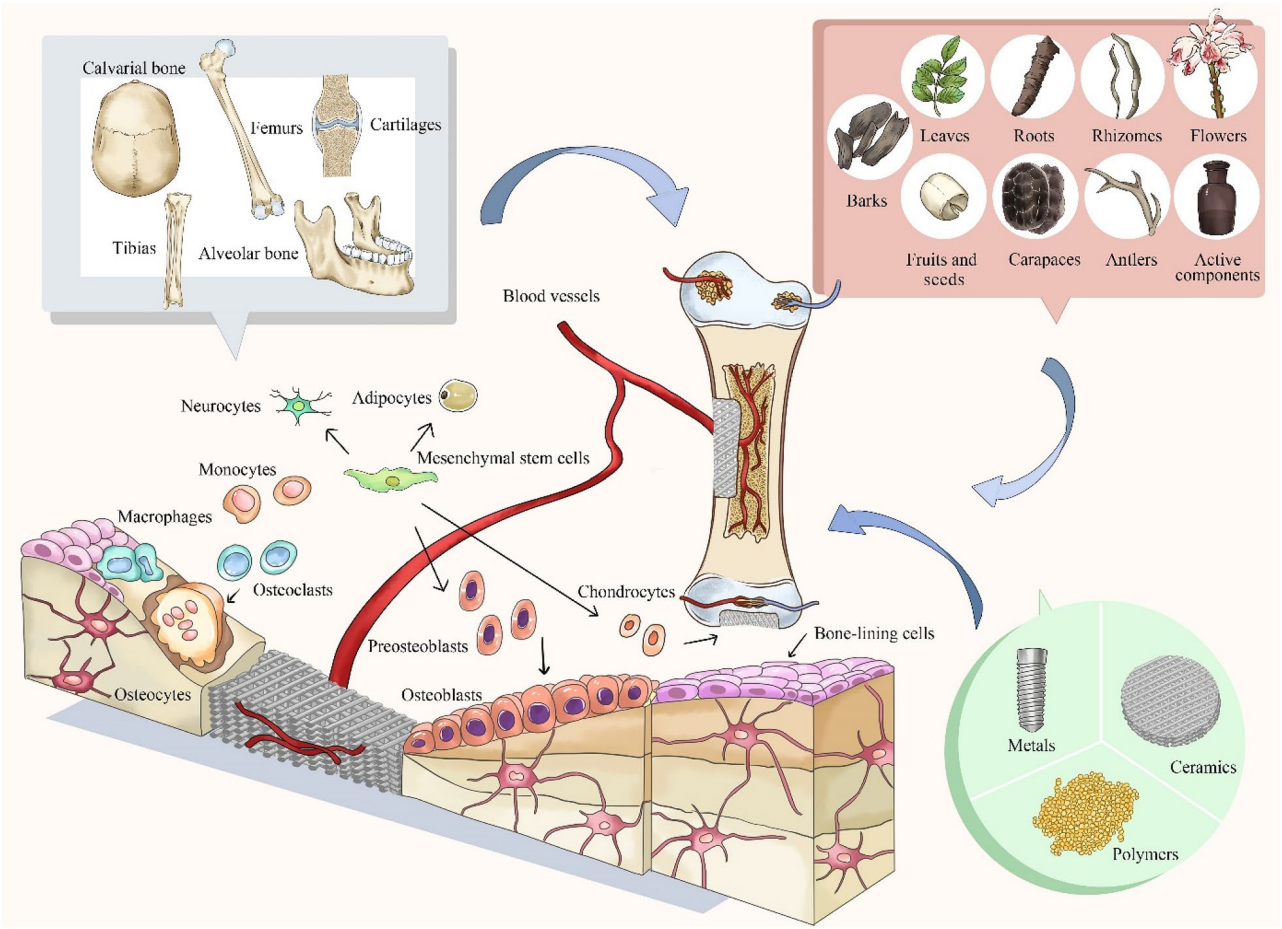
Schematic diagram representing the application of traditional Chinese medicine in bone tissue engineering to accelerate bone regeneration

**Fig. 2:**
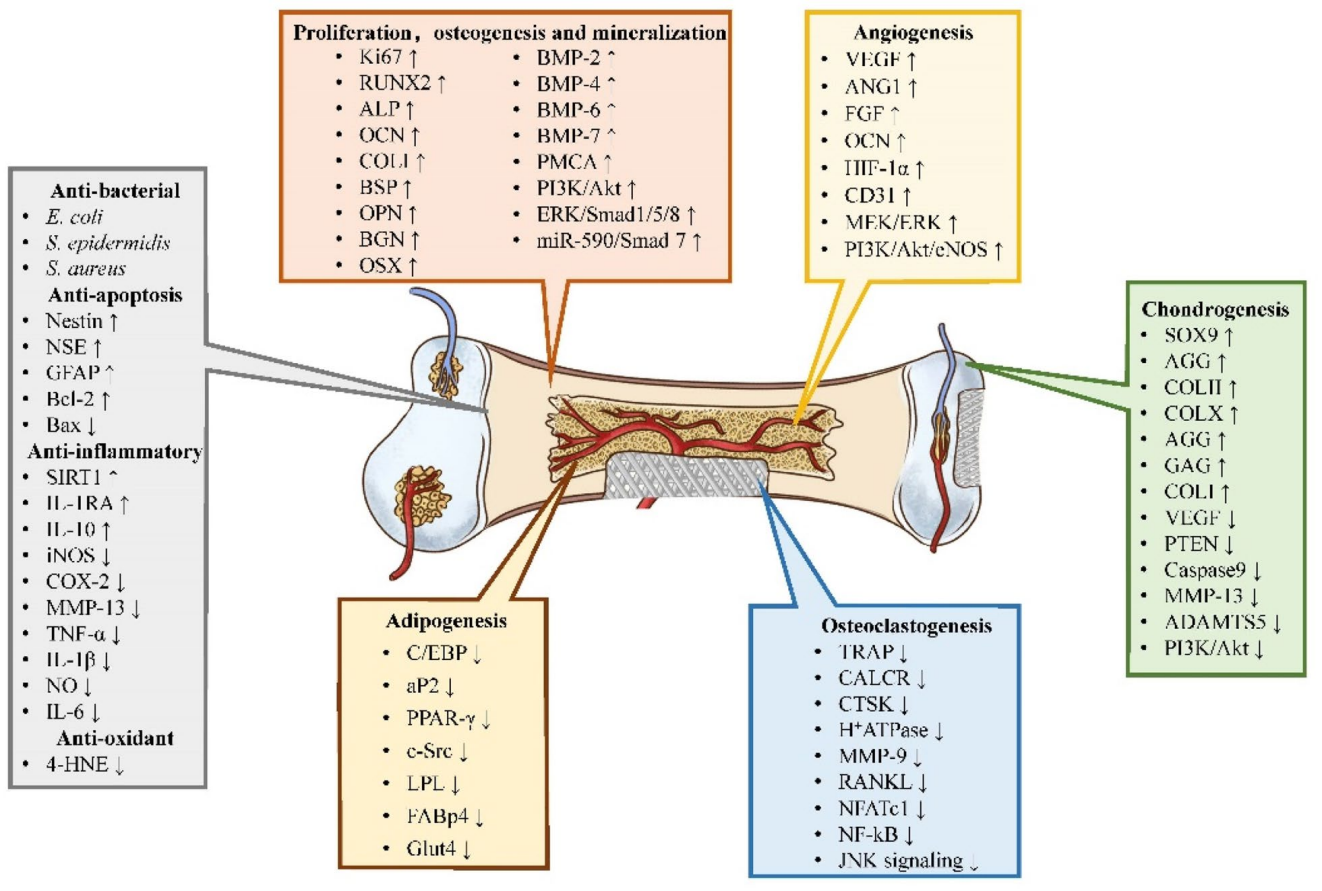
The osteogenic mechanism of TCM ingredients

Bone has the capacity of self-renewal; nevertheless, bone tissue regeneration remains a challenge [77]. The different types of TCM summarized here promote bone formation via multiple mechanisms and targets (Fig. 1), for instance, regulating the process of cell proliferation, osteogenesis and mineralization; chondrogenesis; angiogenesis; osteoclastogenesis; adipogenesis; and anti-inflammatory, anti-oxidant, anti-bacterial, and anti-apoptosis (Fig. 2).

### Proliferation, osteogenesis and mineralization

Traditional Chinese medicine (TCM) has been praised in the world of medicine due to its effects in promoting cell proliferation, regulating bone metabolism, etc. [78], as shown in Table 3. Based on the variety of biomaterials, the use of TCM exhibits great biocompatibility and low cytotoxicity to the cells seeded on the biomaterial, even at extremely high concentrations, as shown by the 3-(4,5-dimethylthiazol-2-yl)-2,5-diphenyl-tetrazolium bromile (MTT) assay and cell counting Kit-8 (CCK-8) assay [76, 79–82]. Icariin loaded PHBV scaffold significantly promoted the proliferation of human osteoblast-like MG-63 cells and the pre-osteoblast MC3T3-E1 cells in a concentration-dependent manner, as shown by Alamar blue assay, and the enhanced cellular proliferation results were due to the upregulating expression of BMP-2, BMP-6, BMP-7 and BGN [83]. Resveratrol and Ang-2 combined with PEGDA/TCS hydrogel showed a good cytocompatibility, and cell density in the resveratrol group was significantly higher than that in the control groups in a hypoxic environment, which was verified by the proliferation marker Ki67 via WB assay [84]. In addition, in the epigallocatechin gallate loaded gelatin scaffold, the chemical modification by epigallocatechin gallate would mitigate MMP-2 and -9 expression, thus slowing down the degradation of gelatin scaffold [85].

Except the low-cytotoxicity of TCM, which provided the basic condition for the application of TCM in BTE, TCM could also directly stimulate osteogenesis. Most studies adopted the rat calvarial bone defect models, while other studies also used tibial plateau defects model, rabbit bilateral thigh muscles model, rabbit lateral femoral condyle model, rat tibial osteotomy model, etc. [86–90]. The results show that more new bone formation and mineralization was found in the center and periphery of the bone defect area after filling with icariin-loaded bioactive scaffold, which attribute to the upregulating of osteogenic-related factors, such as RUNX2, ALP, OCN, COLI, BSP and OPN [76, 86, 91–94]. Besides, other TCM combined with different scaffold materials have similar effects, for instance, icaritin [88, 95], hydroxy safflower yellow A [96], kaempferol [97, 98], naringin [99, 100], quercetin [101–103], silymarin [104, 105], berberine [58, 90, 106], ginsenoside [62, 107], resveratrol [108–110], curcumin [111], and epigallocatechin gallate [112–115]. The rat calvarial defect almost completely repaired with physiological integrity at 16 weeks in the PLGA scaffold incorporated with gelatin, alendronate, and naringin groups, and this might owing to the inhibitory impact of alendronate on osteoclasts and the positive effect of naringin on osteoblasts, the PLGA + Gelatin/ALD/NG scaffold had a high potential for bone repair, as shown by upregulating BMP-2, OSX, OPN, BSP, COLI, OCN and calcium content, and inhibiting TRAP [116]. BMP family possess diverse biological functions during osteogenic differentiation, and including the maintenance of normal bone and bone regeneration. After the treatment of TCM loaded scaffold, BMP-2 and BMP-4 were significantly increased [58, 87, 96, 107, 111, 116–118]. Some of the research has demonstrated the mechanism under the TCM promoting bone regeneration. Baicalin/baicalein loaded Ca-polyP particles rises the intracellular calcium level through activation of the phospholipase C. Meanwhile, both flavones upregulated the expression of the osteoblast calcium efflux channel, the plasma membrane Ca2^+^-ATPase (PMCA), and the expression of ALP, which promotes bone mineralization [119]. Naringin-inlaid composite silk fibroin/hydroxyapatite scaffold had no effect on PI3K and Akt expression but strongly promoted PI3K phosphorylation compared to the control groups, indicating that naringin increased PI3K and Akt activity for stimulating osteogenic differentiation [49]. Using a rat osteotomy model, hesperetin/gelatin sponge scaffold combined with mesenchymal stem cells resulted in accelerated fracture healing, which attributed to the activation of ERK and Smad1/5/8 signaling [89]. Similarly, Zn-silibinin complexes showed promising effects on osteoblast differentiation by regulating miR-590/Smad-7 signaling pathway [120], and ursolic acid loaded mesoporous bioglass/chitosan porous scaffolds would promote the bone regeneration in rat calvarial defect model by stimulating Smad1/5 phosphorylation [118]. Both genipin and proanthocyanidins could act as a cross-linker, and promote the process of osteogenesis via upregulating the expression of RUNX2, OCN, OPN, and ALP [121–124].

**Table 3:** Promoting proliferation, osteogenesis and mineralization

Active components	Biomaterials	Experimental models	Efficacy	References
Icariin	SF/SBA15	Rat BMSC (rBMSC), 38.4 µM	Up-regulating RUNX2, ALP, OCN, and COLI	[94]
	SF/PLCL nanofibrous membrane	rBMSC, 10^−5^ mol/L; rat calvarial defects model	Up-regulating ALP activity	[76]
	PLGA microspheres	rBMSC, 4 × 10^−3^ M; rat calvarial defects model	Up-regulating RUNX2, ALP, OCN, COLI, and OPN	[125]
	Col/PCL/HAp composite scaffolds	rBMSC; rabbit tibial plateau defects model	Up-regulating ALP, COLI, OCN, and OPN	[86]
	CS-modified halloysite nanotubes	hASCs, 10^−5^ M;	Up-regulating ALP	[79]
	CS/nHAP	Osteoblast	N/A	[80]
	PCL/Gel	MC3T3-E1, 0.05 wt%	Up-regulating ALP, OCN, COLI, and calcium content	[91]
	PLGA/TCP	MC3T3-E1, 0.32%; SAON rabbit distal femur defect model;	Up-regulating BSP, OCN, and ALP	[92]
	TCP	Ros17/28, 5 × 10^−5^ M	Up-regulating ALP	[93]
	PHBV scaffolds	MC3T3-E1, MG-63, 25 mg/mL	Up-regulating BGN, BMP-2, BMP-6, and BMP-7	[83]
	BioCaP	MC3T3-E1, 5 mg/L; rat calvarial defects model	Up-regulating ALP, OCN, RUNX2, BMP-2, and COLI	[87]
	ECM-SIS	MC3T3-E1, 10^−5^ M; mouse calvarial defect model	Up-regulating ALP, BSP, OCN, and BMP-4	[117]
Icaritin	PLGA/TCP	Rabbit BMSC, 0.74 g/kg; rabbit bilateral thigh muscles model	Up-regulating ALP and calcium deposition	[88]
	PLGA/TCP	Rabbit BMSC, 1.4 × 10^−3^ M;	Up-regulating COLI, ALP, OCN and calcium deposition	[95]
Hydroxy safflower yellow A	BG	rBMSCs, HUVECs; rat calvarial defects model	Up-regulating RUNX2, OPN, OCN, ALP and BMP-2	[96]
Xanthohumol	HA-g-PLLA	MC3T3-E1, 5, 10, 20 wt%	N/A	[81]
Kaempferol	TiO_2_	rBMSC; rat femur defect	Up-regulating RUNX2, OCN, ALP, OPN and ON	[97]
	Zn	MG-63, 25 µM; Zebrafish model	Up-regulating RUNX2, COLI, ALP, OCN, and ON	[98]
Baicalin and baicalein	Ca-polyP	Primary human osteoblasts, 3 µg/mL	Up-regulating calcium, calcium efflux channel, PMCA and ALP	[119]
Naringin	SF/HAp	hUCMSCs, 0.1 wt%; rabbit femoral distal bone defect	Up-regulating ALP, RUNX2, OSX and COL1A and promoting AKT and PI3K phosphorylation	[49]
	microsphere/SAIB hybrid depots	Primary osteoblasts, 4% w/w; mouse calvarial defect model	Up-regulating ALP, RUNX2 and OCN	[99]
	CS	UMR106, 5 wt%;	Up-regulating ALP	[100]
	PLGA/PLLA/PDLLA	MC3T3-E1, 7 wt%;	N/A	[82]
	PLGA	Rat calvarial defects model	Up-regulating BMP-2, OSX, OPN, BSP, COLI, OCN and calcium content	[116]
Hesperetin	Gel	hMSC, 1 µM; rat tibial osteotomy model	Up-regulating RUNX2, ALP, OCN and COLI and promoting ERK and Smads-1/5/8 phosphorylation	[89]
Quercetin	Ti	hMSCs, 64 ± 10 and 842 ± 361 nmol	Up-regulating ALP activity and calcium content	[126]
	DC/HAp	Rabbit BMSCs, 25 µM; rat calvarial defect model	Up-regulating RUNX2, OCN, and COLI	[101]
	PLLA	MC3T3-E1, 200 µM	Up-regulating RUNX2, ALP, OCN, and COLI	[102]
	MSCS/PCL	Wharton’s jelly MSC, 2%	Up-regulating calcium deposit	[127]
	Decellularized goat-lung scaffold	BMSC, 100 µM	Up-regulating ALP and calcium deposits	[128]
	Ti	hUCMSCs, HGF, 50 nM	Up-regulating RUNX2, COL, OCN, and ALP	[103]
Silymarin	DC/HAp	rBMSCs, 100 µM; rat calvarial bone defect model	Up-regulating RUNX2, COLI, and OCN	[104]
	PLA/Carbon nanotubes	Wharton’s jelly MSCs; rat calvarial bone defect model	Up-regulating ALP	[105]
Silibinin	Zn	C3H10T1/2, MG-63, 60 µM	Up-regulating RUNX2, COLI, ALP, OCN, and miR-590/Smad-7 pathway	[120]
	Alginate/Gel	C3H10T1/2, 20, 50, 100 µM	Up-regulating RUNX2, COLI, OCN, ALP, Pre-miR-20b and pre-miR-410 and down-regulating pre-miR-30c-1and pre-miR-221	[129]
Berberine	PCL/COL	DPSCs, 50 µg/mL; rat calvaria defects model	Up-regulating ALP, BMP-2, COLI and RUNX2	[58]
	PCL/PVP-MC/CS bilayer membrane	MC3T3-E1, 10 µM; rat femur defect model	N/A	[106]
	Negatively charged *O*-carboxymethyl chitosan microspheres	MG-63, rBMSCs; rabbit lateral femoral condyle model	Up-regulating ALP and OCN	[90]
Ginsenoside Rg1	SF/PCL	MC3T3-E1, HUVECs, 5% w/w	Up-regulating ALP, BMP-2, RUNX2 and OCN	[107]
Ginsenoside Rb1	MSCS/PCL	hDPSCs, 5% v/v; rabbit femoral defect model	Up-regulating ALP, OPN and OCN	[62]
Ursolic acid	MBG/CS porous scaffolds	hBMSCs, MC3T3-E1, 5 µM; rat calvarial defect model	Up-regulating ALP, COLI, RUNX2 and BMP-2 and promoting Smad1/5 phosphorylation	[118]
Resveratrol	PEGDA/TCS hydrogel	rBMSCs, HUVECs, 800 µM; rat tibia defect model	Up-regulating Ki67, RUNX2, OPN, and calcium content	[84]
	PLGA microsphere	hMSCs, hTHP-1 monocytes, 25 µM	Up-regulating ALP, OCN and calcium content	[108]
	SLNs/GelMA	rBMSCs, 0.02% w/v; rat calvarial critical-size defect model	Up-regulating ALP, OCN, RUNX2 and OPN	[109]
	PLA/OMMT	hASCs, 0.1 wt%	Up-regulating ALP, OCN and OPN	[110]
Curcumin	PCL	MC3T3-E1, 1 wt%	Up-regulating RUNX2, ALP, BMP-2, OCN, and OPN	[111]
Epigallocatechin gallate	Ti-6Al-4 V	hADSCs, Raw264.7, 0.1, 0.5, 1 mg/mL; rabbit tibias defect model	Up-regulating calcium content, RUNX2, OSX, OCN, OPN	[112]
	PLLA	ADSCs, Raw 264.7, 1 mg/mL; mouse calvarial defect	Up-regulating ALP, RUNX2, and OPN	[113]
	Gel sponges	UMR106, 0.07 mg; rat calvarial defects model	Down-regulating MMP-2, and MMP-9	[85]
	POSS	MC3T3-E1, 6 wt%	Up-regulating ALP	[114]
	DC/HAp	Rabbit BMSC, 5 µM; nude mouse model	Up-regulating RUNX2, OCN, and COLI	[115]
	Gel sponges	Rat calvarial defects model	N/A	[130]
Genipin	CS framework	5 mg	Up-regulating RUNX2, OCN, OPN, and ALP	[121]
	CS	5 mg	Up-regulating RUNX2, OCN, OPN, and ALP	[122]
	Silica particles	20 mM	Up-regulating ALP	[123]
Proanthocyanidins	COLI	10%	Up-regulating ALP	[124]

### Chondrogenesis

Chondrocytes and the surrounding dense layers of extracellular matrix (ECM) form the cartilage [131]. Intramembranous ossification and endochondral ossification are two major processes to form new bone during bone repair [132]. It has been reported that endochondral ossification can be supported by biomaterials, and have a great bone regeneration in clinical [133]. Some of the TCM, for instance, icariin, resveratrol and epigallocatechin, can directly upregulate the expression of chondrogenic-related genes (Table 4). Icariin conjugated hyaluronic acid/collagen (HA/Col) hydrogel showed that the expression of SOX9, AGG, COLII, and COLX was remarkably up-regulated, and the matrix synthesis of sGAG and type II collagen was significantly enhanced [134]. In the rabbit osteochondral defect model, the icariin loaded biomaterials promoting the restoration of supercritical-sized osteochondral defects, as shown by the gross morphology examination and histological analysis, such as hematoxylin and eosin (H&E) and toluidine blue (TB) stained [134, 135]. Besides, icariin has the potential to promote stable chondrogenic differentiation of BMSCs without hypertrophy, and this would further accelerate the process of chondrogenesis [136]. Except upregulating the expression of SOX9, AGG, COLII and COLI in rabbit chondrocyte and BMSCs [137], Yu et al. found that in the resveratrol–PLA–gelatin porous nano-scaffold, the expression levels of SIRT1, AKT and type II collagen proteins was increased significantly, while the expression levels of PI3K/AKT signaling pathway-related proteins, including VEGF, PTEN, Caspase9 and MMP13, was decreased significantly compared to the PLA–gelatin nano-scaffold without resveratrol, which was detected by the immunohistochemical staining. According to the H&E, Masson, Gomori, and Picrosirius red staining, the regenerated cartilage was the thickest, and more chondrocytes were observed with distribution and moderate morphology compared to the negative control groups [138]. The expression of cartilage-specific gene expression, such as s *COLII*, *SOX9* and *ACAN* was detected by Real-time PCR assay, which showed similar trends as biochemical analysis that the epigallocatechin-loaded hyaluronic acid would promote chondrogenesis. COLI and COLX, which are absent in healthy articular cartilage, was also examined and showed lower expression levels of these markers in epigallocatechin-loaded hyaluronic acid group [139].

**Table 4:** Chondrogenesis

Active components	Biomaterials	Experimental model	Efficacy	References
Icariin	HA/Col hydrogel	BMSC, 1 µM; rabbit osteochondral defect model	Up-regulating SOX9, AGG, COL II, and COL X	[134]
	COL	Rabbit chondrocyte, 10^−5^ M; rabbit distal femora osteochondral defects model	Up-regulating AGG, COLII, SOX9, GAG, and COLI	[135]
	Selfassembling peptide nanofiber hydrogel	rBMSC; 1 × 10^−6^ M	Up-regulating COLII and SOX9	[136]
Resveratrol	COLI/PAA	Chondrocytes, BMSCs, 0.5%; rabbit osteochondral defects model	Up-regulating SOX9, AGG, COLII and COLI	[137]
	PLA–Gel porous nano-scaffold	Rat articular cartilage defect model	Up-regulating AKT and COLII and down-regulating PI3K/AKT signaling pathway-related proteins (VEGF, PTEN, Caspase9 and MMP-13)	[138]
Epigallocatechin	Hyaluronic acid	Chondrocytes, 50 µM; mouse osteoarthritis model	Up-regulating GAG, COLII, SOX9 and ACAN and down-regulating ADAMTS5, COLI, and COLX	[139]
Genipin	Carbon dot hydrogel	500 mM	Collagen–genipin–carbon dot nanoparticles improving chondrogenic differentiation and cartilage regeneration	[140]

### Angiogenesis

Bone is a highly vascularized structure. The formation of blood vessel can stimulate and maintain bone cells activity, deliver nutrients and oxygen, and remove metabolites [141]. Notable, a subtype of blood vessel, type H vessels, was provided to be associated with bone formation [142]. TCM would mainly upregulate the expression of angiogenic-related genes, such as VEGF, ANG1, HIF-1α and CD31, to stimulate vascularization Table 5. Liu et al. constructed an osteoporosis model in rats, and the osteogenic and angiogenic differentiation of bone mesenchymal stem cells (BMSCs) treated with icariin was evaluated. Real-time PCR analysis indicated that, similar to the expression of osteogenic genes, the expression of vascular endothelial growth factor (VEGF) and angiopoietin 1 (ANG1) mRNA was promoted by icariin, especially at 20 µM. CPC could act as a suitable icariin delivery system for repairing bone defects, and after implanted into nude mice, the extent of blood vessel growth in the icariin-loaded CPC groups was markedly greater than that in the CPC group. Besides, the systemic administration of icariin has an anti-osteoporotic effect that promotes bone defect repair [143]. Icariin could also induce the osteogenic differentiation in rat ASCs and stimulate the expression of VEGF, which would further promote the process of bone formation, as shown by SEM, micro-CT imaging, H&E and immunohistochemical staining [144]. A phytomolecule icaritin was regarded as a novel osteogenic exogenous growth factor, and a bioactive composite scaffold PLGA/TCP/icaritin was developed. The PLGA/TCP/icariin scaffold was implanted into the bone defect model, and the results of histological staining indicated favorable biocompatibility, rapid bioresorption and more new vessel growth in PLGA/TCP/icaritin scaffolds in contrast to PLGA/TCP scaffolds [145]. Chung BH et al. constructed a similar complex scaffold, and PLGA/TCP/icaritin enhanced new bone formation within the bone defect after core decompression in SAON rabbits and significantly promoted neovascularization in the rabbit muscle pouch experiment, and the mechanism under angiogenesis might due to the upregulating of MEK/ERKand PI3K/Akt/eNOS-dependent signal pathways as previously report [146, 147]. In the rabbit ulnar segmental bone defect model, the blood perfusion within defect sites was detected by dynamic MRI at weeks 2 and 4 post-surgery, which verified the PLGA/TCP/icariin scaffolds induced significant blood vessel ingrowth into the pores of the implanted scaffold in the early stages of bone regeneration compared with that of the control scaffold [148]. Hydroxy-safflower yellow A was loaded into BG scaffolds by coating chitosan/sodium alginate film, and the expression levels of HIF-1α were more pronounced in a dose-dependent manner after 10 days induction in Hydroxy-safflower yellow A loaded groups, as shown by Western blot assay. Hydroxy-safflower yellow A contribute to osteogenesis and angiogenesis definitely, as well as the promotion of repair function and both in vivo and in vitro, the results of high-concentration of Hydroxy-safflower yellow A groups showed the best performance [96]. In the large tibial defect, resveratrol combined with ANG2 could promote new bone formation, and enhance density and size of new blood vessels by increasing autophagy to decrease ANG2 and hypoxia-induced apoptosis, maintaining the growth and proliferation in the endothelial cells, and upregulating the expression of CD31 in the bone defect area [84].

**Table 5:** Angiogenesis

Active components	Biomaterials	Experimental model	Efficacy	References
Icariin	CPC	rBMSC, 20 µM; OVX rat calvarial defect model	Up-regulating VEGF and ANG1	[143]
	SMC-PHBHHx scaffold	BMSC, 10^−6^ mol/L; rat calvarial defects model	Up-regulating VEGF, and FGF	[20]
	45S5 Bioglass	rADSC, 10^−7^ mol/L; rat calvarial defects model	Up-regulating VEGF	[144]
Icaritin	PLGA/TCP	BMSC, BMC 0.052: 100 (powder weight to solution volume); rat calvarial defect model	Up-regulating OCN	[145]
	PLGA/TCP	Rabbit ulnar segmental bone defect	N/A	[148]
	PLGA/TCP	rBMSC, 1 µM; SAON rabbit both distal and proximal femur defect model	MEK/ERK and PI3K/Akt/eNOS-dependent signal pathways	[146, 147]
Hydroxy safflower yellow A	BG	rBMSCs, HUVECs; rat calvarial defects model	Up-regulating HIF-1α	[96]
Silibinin	Zn	C3H10T1/2, MG-63; 60 µM; 3, 7 d	Up-regulating VEGF and ANG1	[120]
Resveratrol	PEGDA/TCS Hydrogel	rBMSCs, HUVECs, 800 µM; rat tibia defect model	Up-regulating CD31	[84]
	PLGA microsphere	hMSCs, hTHP-1 monocytes, 25 µM	Up-regulating VEGF	[108]

### Osteoclastogenesis

The proper balance of osteoblasts and osteoclasts are essential in the maintenance of bone homeostasis [149]. Of these, osteoclasts, derived from haematopoietic lineage, are multinucleated cells involved in bone resorption. Macrophage colony-stimulating factor (M-CSF) and receptor activator of nuclear kappa B ligand (RANKL) is crucial for proliferation and differentiation of osteoclasts, respectively [149]. In the early stage of bone remodeling, osteoclast can remove the dying osteocytes and osteoblasts [150]. This can accelerate the process of bone remodeling. However, the dysregulating between osteoblast and osteoclast would lead to osteoporosis or heterotopic ossification [149]. Icariin and icaritin loaded biomaterials would downregulate the ratio of RANKL/OPG in BMSC and osteoclast, thus promoting osteogenesis by inhibiting osteoclastogenesis [143, 145]. After 7 days of cell culture, osteoclastic markers were evaluated, and quercitrin implant surfaces significantly decreased the expression of osteoclast related genes, including *Trap*, *CalcR*, *Ctsk*, *H*^*+*^*ATPase*, *Mmp9* compared to controls. Besides, the functional osteoclastic markers *Ctsk*, *H*^*+*^*Atpase* and *Mmp9* was significantly lower for quercitrin implant surfaces as well as the expression of *RankL* in vivo [151]. Ursolic acid induced dose-dependent attenuation of titanium (Ti) particle-induced mouse calvarial bone loss, and decreased the number of tartrate-resistant acid phosphatase (TRAP)-positive osteoclasts, which attribute to inhibited the expression of NFATc1 in mRNA and protein level, primarily via the suppression of nuclear factor-kB (NF-kB) signaling, and partly through the suppression of c-Jun N-terminal kinase (JNK) signaling [152]. These results have been shown in Table 6.

**Table 6:** Osteoclastogenesis

Active components	Biomaterials	Experimental model	Efficacy	References
Icariin	CPC	rBMSC, 20 µM; OVX rat calvarial defect model	Down-regulating RANKL	[143]
Icaritin	PLGA/TCP	BMSC, BMC 0.052: 100 (powder weight to solution volume); rat calvarial defect model	Down-regulating RANKL/OPG	[145]
Quercetin	Ti	RAW264.7, 1 mM; rabbit tibia model	Down-regulating Trap, CalcR, Ctsk, H^+^ATPase, MMP-9 and RANKL	[151]
Ursolic acid	Ti particle	RAW264.7, BMMs, 5 µM; mouse calvarial bone defect model	Down-regulating NFATc1, NF-kB and JNK signaling	[152]
Epigallocatechin	Ti-6Al-4 V	hADSCs, Raw264.7, 0.1, 0.5, 1 mg/mL; rabbit tibias defect model	Down-regulating TRAP, CTSK, and RAW264.7 number	[112]
	PLLA	ADSCs, Raw 264.7, 1 mg/mL; mouse calvarial defect	Down-regulating RAW264.7 number	[113]

### Adipogenesis

Numerous studies have indicated a reciprocal relationship between osteoblastogenesis and adipogenesis, and adipogenesis-induction factors would inhibit osteoblastogenesis [153]. However, adipose-derived stem cells (ADSCs), similar to BMSCs, is an immune-privileged cell type with low immunogenicity, and can also differentiate into osteogenic and chondrogenic lineages [154, 155]. The using of icaritin loaded PLGA/TCP scaffold would prevent femoral head collapse in a bipedal SAON model, and this might partly attribute to the inhibiting adipogenic effect of icaritin. In SAON, the expression of adipogenic differentiation regulatory genes C/EBP-β, PPAR-γ and aP2group was control group by 15, 10 and 8 times. After treated with icaritin, the C/EBP, PPAR-γ and aP2 expression was reduced by 72%, 67% and 73%. Of these, C/EBP was required for the downstream proteins involved in adipogenesis, PPAR-γ was the key transcription factor in adipocyte differentiation, and aP2 was regarded as a terminal differentiation marker [156]. icaritin loaded PLGA/TCP also demonstrated the downregulating effect of PPAR-γ in rat calvarial defect model [145]. Cell-infiltratable and injectable gelatin hydrogels would be likely capable of mediating sustained delivery of icaritin to maintain a high-concentration in the long term, and the releasing of icaritin would inhibit the expression of adipogenic markers CEBPα and PPARγ after 7 and 14 days of culture, as shown by Real-time PCR and immunohistochemical staining [157]. The curcumin-loaded silk hydrogel exhibits an interconnected porous structure, and the results of adipogenic markers including PPAR-γ, LPL, FABp4, and Glut4, and the oil red O staining shows that film-associated curcumin accelerates hBMSC adipogenesis when the concentration of curcumin was more than 0.25 mg/mL, while adipogenesis of hBMSCs were inhibited when curcumin concentrations exceeded 5 µM [158]. Madhurakkat et al. found that Epigallocatechin gallate coating on nanofibers can serve as an anti-adipogenic platform by preventing differentiation of ADSCs into adipocytes via inhibiting the expression of LPL and PPAR-γ (Table 7) [113].

**Table 7:** Adipogenesis

Active components	Biomaterials	Experimental model	Efficacy	References
Icaritin	PLGA/TCP	Rabbit BMSC, 3T3-L1, 10^−6^ M; SAON emu proximal femur defect model; SAON rabbit distal femur defect model	Down-regulating C/EBP, aP2, PPAR-γ and lipid droplet	[156]
	PLGA/TCP	BMSC, BMC 0.052: 100 (powder weight to solution volume); rat calvarial defect model	Down-regulating PPAR-γ2	[145]
	Gel hydrogels	hMSC, 100, 200 nM; SAON rat femoral head defect	Down-regulating PPAR-γ and c-Src	[157]
Curcumin	Silk hydrogel	hBMSC; 12.5 µM	Down-regulating PPAR-γ, LPL, FABp4 and Glut4	[158]
Epigallocatechin gallate	PLLA	ADSCs, Raw 264.7, 1 mg/mL; mouse calvarial defect	Down-regulating LPL and PPAR-γ	[113]

### Others

Traditional Chinese medicines exhibit many biological activities, including anti-bacterial, anti-apoptotic, anti-inflammatory and antioxidant effects [159], and these could also contribute to the process of bone formation. Zinc silibinin complex exhibited antibacterial activity in a concentration-dependent manner. There was no significant change in bacterial growth with 1 g/mL of concentration whereas 10 g/mL concentration of Zn-silibinin complexes showed significant against *E. coli* (Gram-negative) and *S. aureus* (Gram-positive) strains compared to control, which would minimize the risk of bacterial infection post implantation and accelerate the augment of bone regeneration [120]. The berberine loaded negatively charged *O*-carboxymethyl chitosan microspheres possessed an ability to reduce the rate of infection caused by *S. aureus*, which can be ascribed to the burst release and diffuse of the berberine [38]. Except process the capacity of promoting osteogenesis, which was verified by the expression level of ALP and OCN, quercitrin-functionalized porous Ti-6Al-4 V implants also presented a great potential in decreasing bacterial adhesion and viability, which could decrease bacterial adhesion by 75% and produce a bactericidal effect [160]. For anti-apoptotic properties, the hBMSCs were co-cultured with ginsenoside Rg1-loaded alginate-chitosan microspheres groups. Ginsenoside Rg1 promotes hBMSC proliferation, accelerates differentiation into Nestin-, NSE- and GFAP-positive cells, and attenuates apoptosis through upregulating anti-apoptotic protein Bcl-2 and inhibiting pro-apoptotic protein Bax compare to the control groups [161]. Resveratrol–PLA–gelatin porous nano-scaffold has been shown to contribute to protect cartilage tissue. This can be attribute to the upregulating of SIRT1, which would delay the MMP13-induced decomposition of cartilage matrix, such as glycogen and II collagen, thus, the life of chondrocytes was prolonged [138].

In the bone defect model, the using of TCM, such as epigallocatechin gallate, resveratrol, ginsenoside Rb1 and baicalin, mainly attenuated the inflammation level by stimulating the expression of anti-inflammatory cytokine IL-10, and inhibiting the pro-inflammatory cytokines TNF-α, IL-1β, IL-6 [62, 108, 112, 114, 139, 162]. Resveratrol was incorporated into atelocollagen hydrogels to fabricate anti-inflammatory cell-free scaffolds, and then, the scaffolds were transplanted into the rabbit osteochondral defects. After implantation for 2, 4 and 6 weeks, the inflammatory related genes IL-1β, MMP-13, and COX-2 were remarkable decreased compared with the untreated defects, as shown by Real-time PCR. After 12 weeks, the osteochondral defects were completely repaired in scaffold groups, which was detected by immunohistochemical and glycosaminoglycan staining [137]. Tetrandrine loaded PLLA films possess sustained releasing behavior. The degree of inflammatory reaction for the implant with the tetrandrine loaded PLLA films was more moderate than control PLLA films in 4, 12 weeks after operation, due to tetrandrine maintained lower levels of inflammatory factors, such as NO, TNF-a, IL-6, iNOS, COX-2, which suggesting that tetrandrine could regulate the mRNA and protein expression to reduce the inflammatory response in macrophages, and accelerate tissue regeneration [163]. Huang AQ et al. combined the well-known antioxidant epigallocatechin gallate into gelatin sponges, and then, the implanted complex would decrease intracellular ROS levels in macrophage cell lines, as shown by anti-4-hydroxynonenal staining, thereby partially inhibiting the expression of MMPs, and promote bone formation (Table 8) [85].

**Table 8:** Others

Mechanism	Active components	Biomaterials	Experimental models	Efficacy	References
Anti-bacterial properties	Silibinin	Zn	C3H10T1/2, MG-63, 60 µM	*E. coli* (Gram-negative) and *S. aureus* (Gram-positive) strains	[120]
Anti-bacterial properties	Berberine	Negatively charged *O*-carboxymethyl chitosan microspheres	MG-63, rBMSCs; rabbit lateral femoral condyle model	*S. aureus*	[90]
Anti-bacterial properties	Quercetin	Ti-6Al-4 V	MC3T3-E1	*S. epidermidis*	[160]
Anti-apoptotic properties	Ginsenoside Rg1	Alginate-CS microspheres	hBMSC, 2 g	Up-regulating Nestin, NSE, GFAP and Bcl-2 and down-regulating Bax	[161]
Anti-inflammatory properties	Resveratrol	PLA–Gel porous nano-scaffold	Rat articular cartilage defect model	Up-regulating SIRT1	[138]
Anti-inflammatory and anti-apoptotic properties	Baicalin	TPGS polymeric micelles	Rat gingival fibroblasts, 20 mg/mL; rat periodontal disease model	Down-regulating TNF-α, IL-1β, and the number of inflammatory cells	[162]
Anti-inflammatory properties	Tetrandrine	PLLA	RAW 264.7, 20 mg; rat model	Down-regulating NO, TNF-a, IL-6, iNOS, and COX-2	[163]
Anti-inflammatory properties	Ginsenoside Rb1	MSCS/PCL	hDPSCs, 5% v/v; rabbit femoral defect model	Up-regulating IL-1RA and down-regulating IL-1β	[62]
Anti-inflammatory properties	Resveratrol	COLI/PAA	Chondrocytes, BMSCs, 0.5%; rabbit osteochondral defects model	Down-regulating IL-1β, MMP-13 and COX-2	[137]
Anti-inflammatory properties	Resveratrol	PLGA microsphere	hMSCs, hTHP-1 monocytes, 25 µM	Up-regulating IL-10 and down-regulating TNF-α and IL-6	[108]
Anti-inflammatory properties	Epigallocatechin gallate	Ti-6Al-4 V	hADSCs, Raw264.7, 0.1, 0.5, 1 mg/mL; rabbit tibias defect model	Up-regulating IL-10 and down-regulating IL-6	[112]
Anti-inflammatory properties	Epigallocatechin gallate	Hyaluronic acid	Chondrocytes, 50 µM; mouse osteoarthritis model	Down-regulating IL-1β and TNF-α,	[139]
Anti-oxidant and anti-inflammatory properties	Epigallocatechin gallate	POSS	MC3T3-E1, 6 wt%	Down-regulating IL-6	[114]
Anti-oxidant and anti-inflammatory properties	Epigallocatechin gallate	Gel sponges	UMR106, 0.07 mg; rat calvarial defects model	Down-regulating 4-HNE	[85]
Anti-oxidant properties	Resveratrol	PLA/OMMT	HASCs, 0.1 wt%	N/A	[110]
Anti-oxidant properties	Quercetin	CS/COL hydrogel	hPDLSC; 100 µM	N/A	[164]
Anti-oxidant properties	Epigallocatechin gallate	PLLA	ADSCs, Raw 264.7, 1 mg/mL; mouse calvarial defect	N/A	[113]

## Limitations, prospects, and conclusions

Although the efficacy of TCM in the treatment of bone regeneration and remodeling has been widely studied, the field is still in its infancy. First, the reliability of TCM is suspected. TCM is a unique Chinese health care system, covering a broad range of medical theories and practices. It has been used for maintaining health and disease treatment over 2000 years. In modern medicine, the use of western medicine has clear indications and contraindications, while TCM is usually applied based on experience. TCM has not be tested by modern scientific research methods, such as cohort studies, randomized controlled studies, and experimental studies. Thus, the safety and effectiveness of their clinical applications lack evaluation. Aristolochic acid has been used in China for hundreds and thousands of years; however, it has been considered as a factor in cancer and kidney damage [165–167]. Although scholars demonstrated that the combined use of berberine reduces the toxicity of aristolochic acid [168], there is still long before aristolochic acid is reapplied in humans for disease treatment. *Areca catechu* L. nut is a well-known traditional herbal medicine, which has been recorded in the pharmacopoeia of China. Areca catechu is known for the treatment of parasitic diseases, dyspepsia, anti-depressant, and anti-migraine effects [169, 170]. However, areca catechu is a group 1 carcinogen and related to oral disorders including submucous fibrosis, oral leukoplakia, and erythroplakia, oral lichenoid lesions, and oral cancer [171, 172].

Second, the mechanism of combining TCM with BTE to promote bone regeneration is in urgent need of further research. Various of TCM have demonstrated, at the cellular level, that they can promote the expression of osteogenic markers. Gavage or intraperitoneal injection in animal models also proved that TCM promotes new bone and blood vessel formation, inhibits osteoclastogenesis, and exhibits anti-inflammatory effects, thereby promoting the process of bone remodeling and regeneration. Schisandrin A, isolated from *Schisandra chinensis* (Turcz.) Baill, is a promising medicine for osteoporosis treatment by inhibiting RANKL-induced ROS via the overexpression of nuclear factor erythroid 2-related factor 2 (Nrf2), suppressing the differentiation of osteoclasts [173]. Gastrodin, extracted from *Gastrodia elata* Blume, could reduce IL-1β-induced apoptosis in chondrocytes and attenuate the release of inflammatory mediators IL-6 and TNF-α, thus ameliorating rat cartilage degeneration [174]. Morin, a flavonoid derived from old fustic and osage orange trees, stimulates the Wnt pathway by activating and translocating of β-catenin nuclei, thus promoting osteoblast development [175]. However, the efficiency of TCM in bone regeneration must be further verified, as they may not play the same role in BTE. *Salvia miltiorrhiza* belongs to the *Lamiaceae* family and is mainly applied to enhance blood circulation and for cardiovascular disease treatment [176, 177]. Because of its pharmacological properties, *Salvia miltiorrhiza* not only promotes bone formation by regulating ALP, OCN, OPG, and RANKL expression, but also by stimulating angiogenesis via upregulation of the expression of VEGF [177]. In the *Salvia miltiorrhiza*-coated Ti sample, little apatite was formed on the surface, which was not significantly different from the control group [178].

Third, most research was conducted on plant-derived phytochemicals, while the proteins and peptides, both plant- and animal-derived, have not been thoroughly researched due to large quality difference from batch to batch. Although the development of recombinant technology can solve this problem to a certain extent, few proteins and peptides have been investigated [179]. Ling Zhi-8, purified from *Ganoderma lucidum*, is an immunomodulatory protein consisting of 110 amino acid residues. Ling Zhi-8 is a promising anti-osteoporosis drug for both preventive and therapeutic effects, which can regulate RANK/RANKL/OPG signaling and inhibit the level of c-Fos and NFATc1, two key target genes of the osteoclast [180, 181]. In a standardized nasal bone defect, the polyurethane (PU)-based material was filled, and a higher bone volume was observed in the Ling Zhi-8-treated sample, although it was not as strong as BMP-2-treated sample [182]. Thus, the protein and peptide related research is promising. Not only proteins, but generally animal-derived TCMs were largely neglected due to slow and expensive research procedures, unstandardized material bases, and unclear active components [12]. Some of the animal-derived TCMs have been applied in BTE, such as *Colla Cornus Cervi* and *Colla Plastri Testudinisis*. Further, other animal-derived TCMs may also have potential in bone regeneration. Kangfuxin, extracted from *Periplaneta americana*, has been verified in the mechanism against osteoporosis, as it accelerates bone formation through stimulating osteoblasts and HUVECs activities, while decreasing bone absorption by inhibiting osteoclast activities [183]. The kangfuxin-coated alginate/carboxymethyl CS sponge exhibits excellent antibacterial, cytocompatibility, and rapid hemostasis effects, and stimulates wound healing [1]. Furthermore, there are even fewer studies on mineral-derived TCM in bone regeneration. Therefore, TCMs that we investigated are only the tip of the iceberg, and which of them may become the “artemisinin” in bone tissue engineering is subject to further research.

With the modernization of TCM and the development of analytical and detection techniques, TCM has gradually transformed from an experience-based to an evidence-based medicinal system. The combination of TCM with “-omics”, such as metabolomics [184], microbiome [185], proteomics [186], and herbgenomics [187], can elucidate the mechanism and molecular targets of TCM, and further promote the development of TCM in the direction of precision medicine. In summary, TCM is a promising therapeutic method both in bone regeneration and BTE. This review provides a reference for the research of TCM for the application in BTE.
